# Memoir on Softening of the Stomach in Infants

**Published:** 1829-10-01

**Authors:** 


### XLII.

### A MEMOIR ON GASTROMALAXIA, OR SOFTENING OF THE STOMACH IN INFANTS. By FRED. FERDI FELS, M.D. of Leipsig. Translated by M. GENDRIN.

We have more than once noticed this disease in former numbers of the Journal; but the memoir of Dr. Fels, and the notes of M. Gendrin, present the fullest account of the malady which has yet appeared in any language. We therefore deem it necessary to lay a pretty copious analysis of the work before the British profession.

Gastromalaxia is a disease of infancy which is, as yet, but little known. It is, as the name imports, a softening of the stomach. Custom has led physicians to name and characterise a disease by the effects or consequences of that disease, as seen in the dead body. This is illustrated by the malady under consideration, which, like most others, is determined by general causes, but produces local lesions of certain organs or parts, by which life is too often destroyed. The disease in question has received different names from different writers. Goedecke calls it a *dissolution* of the stomach—Hildebrand a *softening*—Laisné, an *erosion and spontaneous perforation* of the organ. The perforation is clearly a consequence of the softening—and death often takes place before the perforation occurs. We agree with the author that all names involving the supposed nature or causes of a disease, should be avoided, as liable to fluctuation and error. The actual appearances in the body, either before or after death, when they are at all uniform, afford a surer basis for nomenclature.

Gastromalaxia, like most other diseases, is either simple or complicated—acute or chronic.

I. *Simple Acute Gastromalaxia.* After some days of moroseness and loss of appetite, sometimes suddenly and without premonitory symptoms, the disease sets in with insatiable thirst, red and sometimes aphthous tongue, abdomen often soft and compressible, but generally tender on pressure, especially at the epigastrium, where the temperature is usually higher than elsewhere. Vomiting is sometimes a very early symptom—at other times it comes on later. It is always aggravated by the ingestion of either liquids or solids. The vomiting alternates or coincides with diarrhœa, which is of a watery or porraceous kind. The respiration is usually short and quick, interrupted by a dry cough. The infant cries constantly, and apparently from pain. The face is alternately pale and flushed, the transitions being very rapid, and the countenance always bearing the indications of depression and moroseness. The eyelids are red, contracted, and sometimes œdematous—agitation of the limbs constant—temperature of the surface somewhat elevated—fever sometimes violent, at others, equivocal, the pulse being quick, unequal, or intermittent. The vomiting and diarrhœa continue—the cries are changed into moanings—there is occasional coma, from which, however, the patients are easily roused—they lie on their backs, the legs drawn up—the extremities get cold—the eyes are turned up, and the eyelids half closed—the face becomes Hippocratic, and the patients die either in convulsions or without any apparent commotion. The acute form of the disease usually runs its course in forty-eight hours—and is rarely prolonged to the sixth or eighth day.

II. *Chronic Gastromalaxia.* This form generally runs two periods. The *first* sometimes lasts several weeks, and is characterised by general symptoms:—the *second* presents the characters proper to gastric affection. The duration of this period is usually only a few days.

*First Period.* The disease frequently occurs at the time of weaning. The child becomes morose, loses its appetite—the abdomen is tense, but not always tender on pressure—the digestion is deranged, as indicated by watery, green, or other morbid stools—there is occasional vomiting, apparently excited by a dry cough—emaciation takes place rapidly—the fever, when it exists, is by no means strong. In proportion as the disease advances the little patient refuses food, but sucks if at the breast, or craves for drink if weaned.

*Second Period.* This is manifested by symptoms directly indicative of gastro-malaxia. The vomiting occurs without any food or drink having been given—the diarrhœal discharges emit a very fetid odour. There is sometimes obstinate constipation. The belly becomes tense—the tongue red and furred—knees drawn up towards the abdomen. We do not, however, see by our author's description that there is any very marked symptom distinctive of the two periods.

III. *Complications.* The disease in question may be complicated with encephalitis, acute hydrocephalus, pneumonia, abdominal inflammations, dysentery, intestinal worms, intermittent fevers, and many of the eruptive diseases.

APPEARANCES ON DISSECTION.

In those who have died of gastromalaxia, we sometimes find the stomach distended with gas, sometimes with fluids or solids. Occasionally the organ is found burst—but more frequently a change in the textures of the viscus only is observed. The most common seat of the disease is in that part of the stomach which is next to the spleen. The pyloric region is rarely affected. The softened membranes may be increased in thickness, diminished, or of their natural size. The softening is sometimes confined to the mucous membrane—sometimes it extends to the whole of the coats, even to the peritoneal.

The author describes three degrees of intensity in this morbid ramollissement. In the first degree the texture of the tissues is not altered, but the membranes are flabby, soft, and easily torn. At a more advanced period the membranes are seen to be changed into a gellatinous substance, but still shewing traces of organization. The parts are easily mashed between the fingers, and the softened membranes can be readily wiped away even by a sponge. In the greatest degree of the disease there is no remaining vestige of original texture or organization, the membranes being converted into a homogeneous, pultaceous substance, through which one or more perforations have taken place, with fringed and softened borders.

The colour of the membranes in this disease is various, and sometimes little different from the natural tint. The same may be said of the extent to which the disease spreads. It is very various. For the most part there are evident traces of inflammatory action in the other parts of the stomach, or near the softened portions. The contained matters, in such circumstances, are generally very acid, and but rarely putrid.

### Etiology.

Gastromalaxia may be considered as peculiar to infancy—being rarely seen after the age of two years. It generally shews itself during the first dentition, and especially at the period of weaning, when the child begins to exchange the milk of the mother for more solid nutriment. The author enters into some speculations on the reasons why infants should be more subject to the disease than adults; but we do not deem it necessary to follow him in this part of the investigation. Among the predisposing causes may be ranked all those which are capable of deranging the digestive functions. The unwholesome milk of a nurse—too early weaning—aliments of difficult digestion—the application of cold to the surface—the abuse of stimulants and narcotics—various diseases, as whooping-cough—worms—intermittent fevers, and the eruptive diseases, &c.

### Diagnosis.

The following are considered as the most pathognomonic symptoms; viz. diarrhœa, consisting of copious watery, mucous, and greenish stools—bilious vomitings, indomitable by any remedy—ardent thirst—irregular fever—prostration of strength—drawing up of the feet, and especially of the left foot—paleness and coldness of the extremities—rapid emaciation of the whole body, but especially of the neck—soporose state, from which the patient is not easily roused, the cerebral functions not appearing to be much affected—stifled groaning—face expressive of great distress. At the same time it is necessary to state that it is by a reunion of several of these symptoms that we can hope to recognise the disease—and not by any one of them.

In respect to the proximate cause of the disease we fear that very little satisfactory can be adduced. The opinions of Hunter, Cruickshank, Heischmann, Cruveilhier, and many others, are canvassed, but without benefit. When the author himself says that the proximate cause of gastromalaxia may be a loss of vital energy in the organ, he offers us a vague idea, of which we can make little or no use.

The dangerous tendency of the disease is unquestionable. Not more than one out of eight or ten infants, he thinks, are preserved, when this malady has once commenced. Of the treatment, too, we have little consolatory, and nothing positive, to put on record. Several physicians, and, among others, Massius, Pohli, and Gairdner, have recommended the antiphlogistic treatment. M. Cruveilhier, who appears to have had most success as well as experience in this complaint, strongly *advises* us to put the child on a system of starvation, not only as to food, but as to drink. He prohibits all kinds of sustenance, but barley water mixed with some yolk of egg, and given in the quantity of a table-spoonful every two, three, or four hours. To children recently weaned he gives small quantities of the nurse's milk. He prescribes tepid baths from the beginning—but his sheet-anchor is opium, given both by the mouth and by injection. This administration of opium is censured by many writers; but almost all agree in recommending rigid abstemiousness, the tepid baths, and abstinence from all purgatives. For our own parts, without being able to speak from much experience in this rare and dangerous malady, we would, from reasoning on the data actually on record, regarding its pathology, be inclined to trust mainly to the powers of Nature, taking care not to interfere with these powers by any active medicine exhibited by the mouth. Arrow-root and milk appear to be the best kind of nourishment, keeping the bowels clear by means of watery and oily injections. If, as is not improbable, there be often some inflammatory action at the bottom of the malady, there can be little danger, and there may be some benefit, in the application of leeches to the epigastrium, and even some light counter-irritation there afterwards. Considering the occasional acrimony of the secretions in the first passages, at all periods of life, even the earliest, we conceive that absorbents are strongly indicated in this disease. We should be inclined to exhibit almond emulsion with magnesia and small doses of hyosciamus, as soon as the symptoms are strongly marked, and after the bowels are cleared by castor oil.

To the Essay in question is appended a considerable number of cases, many of them interesting and instructive. The learned editor of the *Journal General de Médécine*, (M. Gendrin) has also added a case that happened under his own observation, accompanied by some practical remarks, which are deserving of attention. For these, however, we must refer to M. Gendrin's translation of the essay, as published in the 8th volume of the 3d series of the above journal.

---

